# Controls on eDNA movement in streams: Transport, Retention, and Resuspension

**DOI:** 10.1038/s41598-017-05223-1

**Published:** 2017-07-11

**Authors:** Arial J. Shogren, Jennifer L. Tank, Elizabeth Andruszkiewicz, Brett Olds, Andrew R. Mahon, Christopher L. Jerde, Diogo Bolster

**Affiliations:** 1 University of Notre Dame, Department of Biological Sciences, Environmental Change Initiative, Notre Dame, Indiana USA; 2 University of Notre Dame, Department of Civil and Environmental Engineering and Earth Sciences, Notre Dame, Indiana USA; 3 Stanford University, Department of Civil and Environmental Engineering, Stanford, California USA; 4 Hawai’i Pacific University, Shrimp Department, Oceanic Institute, Waimanalo, Hawaii USA; 5 Central Michigan University, Department of Biology, Institute for Great Lakes Research, Mount Pleasant, Michigan USA; 6 Marine Science Institute, University of California, Santa Barbara, Santa Barbara, California, USA

## Abstract

Advances in detection of genetic material from species in aquatic ecosystems, including environmental DNA (eDNA), have improved species monitoring and management. eDNA from target species can readily move in streams and rivers and the goal is to measure it, and with that infer where and how abundant species are, adding great value to delimiting species invasions, monitoring and protecting rare species, and estimating biodiversity. To date, we lack an integrated framework that identifies environmental factors that control eDNA movement in realistic, complex, and heterogeneous flowing waters. To this end, using an empirical approach and a simple conceptual model, we propose a framework of how eDNA is transported, retained, and resuspended in stream systems. Such an understanding of eDNA dispersal in streams will be essential for designing optimized sampling protocols and subsequently estimating biomass or organismal abundance. We also discuss guiding principles for more effective use of eDNA methods, highlighting the necessity of understanding these parameters for use in future predictive modeling of eDNA transport.

## Introduction

Streams and rivers connect landscapes to oceans, moving natural and anthropogenic materials through extensive and heterogeneous network systems^1, 2^. One substance that is actively transported in these lotic ecosystems that has rapidly gained interest is environmental DNA, hereafter eDNA^3, 4^. In aquatic systems, eDNA consists of genetic material in the form of free DNA, mucus, urine, blood, feces, cells, and tissue that is released into the water column^3–6^. To date, the use of eDNA detection techniques have been a powerful tool for discovery, surveillance, and monitoring of endangered^7^, rare^8^, and invasive species^3, 4, 9^. In standing water (i.e., lentic) systems like lakes and ponds, eDNA has been used to estimate population abundance of target species^10, 11^. Yet in flowing (i.e., lotic) waters, progress has been limited to various studies that produced varied results with regard to instream eDNA transport and linking to community structure (e.g., refs 12–14). However, to date we have not identified all environmental factors that control species eDNA movement and fate in realistic and heterogeneous flowing environments.

Modeling transport of any substance in stream and river environments is challenging. Conceptually, natural hydrologic complexity is often over-simplified and streams are treated as conduits that move water and material downslope^15–17^. In reality, streams are complex, reactive systems, with rapid, turbulent flows connected to slower flows in the subsurface and near-surface streambed^1^. These interactions cause solutes and particles to leave the main flow and either be temporarily retained or entirely removed by physical storage and retention^1, 18–20^. or biological removal^21, 22^. In-stream complexity depends on flow structure and the makeup of the streambed^18, 20, 23^. Thus, increasing evidence highlights how physical complexity of streambeds exerts strong controls on ecological processes^24^. Understanding the physical and biological variables that influence the retention and transport of substances in flowing waters is essential for modeling efforts in streams and rivers^25^.

In addition to flow characteristics, the unique physio-biogeochemical properties of the substance, here eDNA, that is being transported will likely influence persistence, degradation, and ultimately detection of species^26^. We know eDNA is polydisperse^27, 28^, as it is made up of variably sized particles from a variety of source materials (i.e., cells, tissue, feces, mucous, etc.) that can range broadly in their transport behavior^13^; this unique characteristic of eDNA introduces an additional level of complexity to an already complex problem^12, 26^. Previous studies have shown that eDNA can be transported over long distances in flowing waters^14, 29–31^, but has been limited to using eDNA as a qualitative detection tool for confirming presence-absence of a species *somewhere* in the fluvial network. There remain very few investigations focused on the mechanistic details of how genetic materials are transported or retained within flowing waters; previous studies have hypothesized the mechanisms for persistence and transport of eDNA^30^, but to date have found weak relationships between population abundance and downstream eDNA concentrations^32^. Describing the controls on transport and retention of eDNA will be critical in order to uncover where, when, and how many individuals exist in a fluvial system.

Recently, a more mechanistic understanding of eDNA transport has begun to emerge, built on eDNA releases into experimental streams. Importantly, this understanding reflects three major transfer mechanisms in streams: transport (downstream movement driven by bulk water flow^30, 32^) retention (deposition or capture by the streambed^13^), and resuspension^12^. As a critical first step in putting these three factors together, Jerde *et al*.^12^ found that in a stream environment, eDNA will be significantly retained by the streambed as it is transported, its concentration decreasing with downstream distance. Additionally, while much eDNA is deposited on the streambed, some will be later resuspended, though these events are inherently difficult to predict. One study^12^ provided a first step in demonstrating the importance of instream retention in controlling eDNA detection from samples taken from the water column, but the estimation of transport and deposition rates was constrained by low eDNA concentrations, and high measurement variability related to the polydisperse nature of eDNA. Additional work^13^ showed that fine substrate (sand) would retain more eDNA than course substrate (pea gravel). Thus, there remains a need for an improved understanding of interplay between critical variables controlling in-stream transport, retention, and resuspension of eDNA materials, which will ultimately facilitate quantitative interpretation, prediction, and modeling efforts of eDNA transport in fluvial systems to better predict the upstream presence of eDNA detected species. Parameterization of such mechanisms would move us from qualitative detection toward quantitative understanding and prediction of location and estimation of abundance, and will be critical in taking eDNA detection techniques to the next level as a powerful diagnostic tool in ecology, conservation, and management.

In this study, we address three main processes controlling eDNA movement in streams: 1) *Transport:* How far does an average eDNA particle travel in streams with different hydrologic signatures and how might eDNA detection be used to infer where a species is located?; 2) *Retention:* Does surface-subsurface exchange trap eDNA in porous, benthic substrate interstices and does the presence of biofilms of organic matter play a role?; and 3) *Resuspension:* Can resuspension from the streambed result in eDNA detection after the source of eDNA has been removed, and does this depend on streambed characteristics? To answer these questions, we conducted a series of experimental eDNA additions into four experimental streams that varied only in the substrate lining the bottom. Due to differing substrate size and structural complexity, each stream exhibits unique hydrologic and transport properties, with a signature residence time distribution (RTD), controlled by how long solutes reside in the “slow” streambed, and stream-to-bed exchange rate, representing how quickly and how much water is exchanged between stream and streambed^20^. Both RTD and exchange rates are known to be important in conservative transport; we suggest that they are also important controls in eDNA movement.

**Figure 1:**
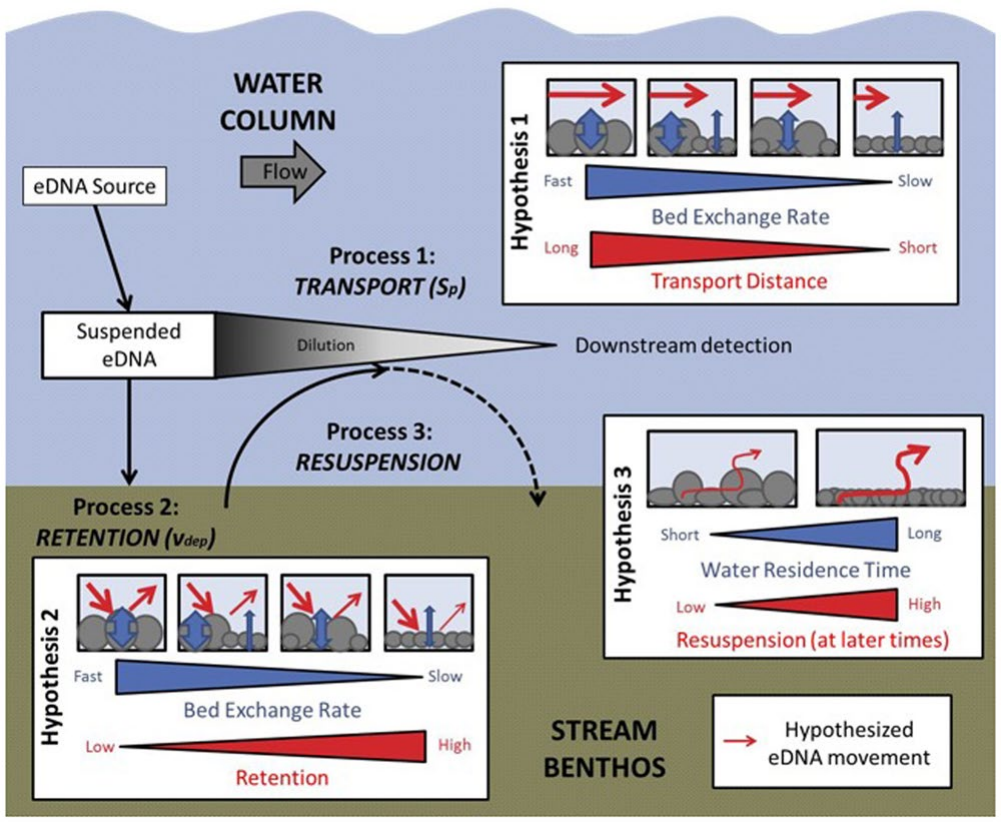
Conceptual diagram depicting the three governing processes of eDNA movement in streams: 1) Transport, 2) Retention, and 3) Resuspension, and associated hypotheses for eDNA movement.

To answer the above questions, we propose three hypotheses: 1) *Transport* downstream of eDNA will be limited by hyporheic exchange rates (i.e., how quickly water moves in and out of the streambed); 2) *Retention* and deposition of eDNA will be controlled by the rate of hyporheic exchange, but limited by substrate size, with smaller substrates retaining a larger fraction of eDNA; and 3) *Resuspension* of eDNA during transport will “spiral” as it moves downstream via alternating deposition and resuspension events, resulting in “power law tailing” behavior as the source of eDNA is removed from the water column. Our three hypotheses are highlighted schematically in Fig. 1. We structured our experiment to estimate eDNA RTDs for each stream, given the importance of parameterizing how far an eDNA particle could travel before permanent removal (e.g., via degradation, consumption, or sorption). Using data from our experimental eDNA additions, we integrate these three questions and provide suggestions for field sampling for eDNA sampling in natural systems, especially for linking eDNA concentrations with estimation of biomass and/or abundance.

## Methods

### Site description

**Figure 2:**
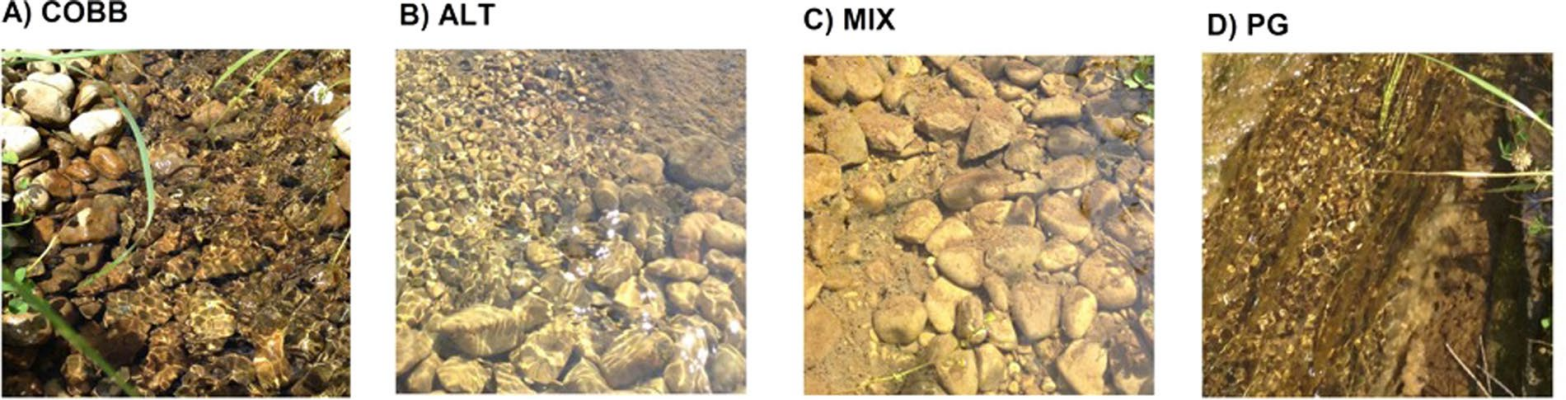
Pictures of COBB (**A**), ALT (**B**), MIX (**C**), and PG (**D**) ND LEEF streams in August 2014, after ~60 days of biofilm accumulation.

We conducted this experiment in 4 streams at the University of Notre Dame Linked Experimental Ecosystem Facility (ND LEEF) in the summer of 2014. The streams are 0.4 m wide, 7–10 cm deep, and 60 m long, and stream water is sourced from a constant-head reservoir fed by oligotrophic groundwater. All four streams are identical in all aspects, except that they each have a unique benthic substrate lining the stream bottom: each configuration represents a different combination of substrate size and structural complexity. Each stream was lined with a unique configuration of substrate: pea gravel (PG, D_50_ = 0.5 cm) and cobble (COBB, D_50_ = 5 cm) and structural complexity: alternating 2 m sections of PG and COBB substrates (ALT) and a random 50/50 mix (MIX) (Fig. 2). Flow in all 4 streams is maintained at ~2 L/s, and before beginning our experiments, we allowed all streams to naturally colonize with biofilm and periphyton for ~60 days. Previous eDNA studies in these streams released low concentrations of eDNA from bluegill (*Lepomis macrochirus*) and largemouth bass (*Micropterus salmoides*) in the summer of 2013^12^; here we used higher concentrations from common carp (*Cyprinus carpio*).

### Quantifying biofilm growth

Before each eDNA experiment, we collected substrate samples in 160 mL specimen cups for estimation of autotrophic biofilm using chlorophyll *a* and organic matter content (expressed as ash-free dry mass; AFDM) by sampling every 5 m along the reach; n = 10 samples per stream). On half of the samples, we extracted chlorophyll *a* and measured it fluorometrically using standard methods^33^, then corrected for surface area of each substrate sampled. On the other half, we estimated AFDM by placing the ~20 mL of substrate into 100 mL of water and mixing vigorously to loosen biofilm from the substrate. We filtered slurry onto a pre-ashed and weighted GF/F (Whatman) filter, dried for 48 hrs at 60 °C, measured dry mass, then ashed at 550 °C for 1 hr, re-wet, re-dried for 48 hours at 60 °C, and measured ashed mass, with difference representing AFDM^33^.

### Steady state releases to estimate transport and retention

For all experimental additions, we injected an eDNA solution at the top of each stream reach that consisted of DNA collected from a water source in a local fish pond (South Bend, IN, USA) containing a high density of common carp (*C. carpio*). Our experimental protocol was modified from a common approach used to estimate nutrient transport^34^. Using standard methods for short-term solute additions, we measured reach-scale retention of eDNA during short-term (4 hr) additions^35^. Prior to the addition of eDNA, we collected 5 samples (250 mL each) of ambient stream water along each study reach (at 10, 20, 30, 40, and 48.5 m downstream from release site). The streams were fed by a groundwater-holding reservoir containing no observable fish, although we used these field blank samples for additional quality control and confirmation.

To begin the experiment, we added the common carp eDNA solution at the head of each stream at a rate of 100 mL/min using a battery-operated pump (Fluid Metering) to elevate in-stream concentrations. We simultaneously added a conservative tracer (Cl^−^ as NaCl) into the stream to account for any dilution and the injection location had four baffles to ensure full mixing of the release solution into the streamflow. This phase of the experiments represents a steady source of eDNA (e.g., a fish) being released at the head of the stream, which is then transported downstream with flow. After 4 hrs of steady state release of eDNA solution into each stream, conservative tracer concentrations were uniform throughout the entire reach, indicating that streams had reached a completely mixed plateau phase. At the 4-hour mark, we collected three 250 mL replicate water samples longitudinally at each of the five measurement sites (n = 15 samples per stream). To account for any possible degradation that had occurred over the initial 4 hrs, we took replicate 250 mL samples from the eDNA release solution bucket (n = 5) every 30 min during each release. We placed all water samples immediately on ice in a bleach-sterilized cooler and filtered them within four hrs of collection. We also placed four 250 mL bottles filled with deionized water in the cooler to serve as field controls (“cooler blanks”).

### Post-sampling after eDNA source removal to estimate resuspension times

In each stream, after the plateau samples were collected, we turned off the head pump supplying the eDNA solution, thus removing the source of eDNA (i.e., simulating fish removal). In order to estimate residence time of eDNA in our stream reaches, we then sampled the “falling limb” (i.e., concentration over time after the pump is turned off) of eDNA at the station furthest downstream (48.5 m). As soon as the pump was turned off, we took 250 mL samples at the following time intervals to quantify the total residence time of eDNA in each stream: at 5-minute intervals for 50 minutes (n = 10), at 15-minute intervals for an hour (n = 4), and then at 30-minute intervals for 2 hours (n = 4) until the 4 hr time point was reached (n = 18 total samples). This phase of the experiment measured resuspension of any short-term retention of eDNA and represented “flushing” of eDNA mass from each stream.

### Sample Filtration and Extraction

We vacuum-filtered all samples through 1.2 µm Isopore™ polycarbonate membrane filters (EMD Millipore Corporation, Billerica, MA, USA) using sterile 300 mL filter cups. We isolated eDNA from filters by combining the polycarbonate membrane filter with 700 *µ*L of CTAB Buffer and 20 *µ*L of proteinase K, vortexed for 15 sec, and incubated at 63 °C for 2 hrs. After incubation, we added 700 *µ*L of a 24:1 chloroform:isoamylalcohol solution to the solution and vortexed for 5 sec, centrifuged at 15,000 RPM for 10 min, and transferred 500 *µ*L of the supernatant to a new tube. Next, we added 500 *µ*L of isopropanol and 250 *µ*L of NaCl, inverted the sample gently to mix, and then incubated at −20 °C for ≥4 hrs. We removed samples from −20 °C, centrifuged at 15,000 RPM for 10 min to form a pellet, poured off the supernatant, and washed twice with 150 *µ*L 70% ethanol, pouring off ethanol and maintaining the pellet each time. We dried the pellets 15 min at 45 °C in a vacuum centrifuge and then resuspended in 200 *µ*L TE buffer overnight at 4 °C in a refrigerator prior to qPCR. For every 20 samples, we added a filter-less extraction blank (i.e., tube with all reagents) to serve as a lab control. Filtration and extraction methods above followed detailed protocols described by Renshaw *et al*.^36^.

### eDNA quantification

We assayed all DNA extractions with qPCR TaqMan® primers and probe in the following 20 µl mixes: 10 µl of TaqMan® Environmental Master Mix 2.0 (Life Technologies), 1.8 µl of each primer (900 nM well concentration), 0.25 µl of the TaqMan® probe (125 nM well concentration), 4 µl of extracted DNA, and 2.15 µl of sterile water. We used primers specific to *C. carpio* from the mitochondrial cytochrome *b* gene (as described in 10). We used the following cycling parameters: a single step at 50 °C for 2 minutes, a single step at 95 °C for 10 min, and 55 cycles at 95 °C for 15 sec followed by 60 °C for 1 min. To quantify the DNA copy number in each DNA extract, we created a synthetic standard and included it on each qPCR plate along with the DNA extracts^10^. We determined the copy number of the synthesized standard by dividing the molecular weight by Avogadro’s number. We ran a serial dilution of the standard on each qPCR plate and provided a regression line from which the unknown copy numbers of the DNA extracts could be estimated, along with a non-template control to assess contamination. We ran all qPCR assays on a Mastercycler ep realplex real-time PCR system (Eppendorf) and analyzed with Realplex 2.2 software, running each sample in triplicate. While we did not directly test for qPCR inhibition using an internal positive standard, we ran all samples using a commercially available Environmental MasterMix, which has been found to significantly reduce the effects of inhibition on environmental samples^30^. All field, cooler, and extraction negative controls, which were treated in the same manner as field samples, and all qPCR NTCs tested negative for carp eDNA and the standard curve efficiency ranged from 95% to 98%, and R^2^ ranged from 0.96 to 1.00. Based on standard curve amplification, the 95% limit of detection (LOD) was 30 copies per reaction, and the lowest concentration standard (3 copies per reaction) amplified 70% of the time.

We expected that the concentrations of eDNA in the “falling limb” samples would be low, so we analyzed them using digital droplet PCR (ddPCR). At higher concentrations (>200 copies/μL), recent research has shown that qPCR and ddPCR are equivalent at detecting eDNA^37^ however, ddPCR can detect eDNA significantly better at very low concentrations (<3 copies/mL)^12, 25, 38^. Using the same primers and Taqman probe from qPCR, target DNA was randomly allocated on to discrete droplets using microfluidics and then each of the samples, containing 10,000 to 20,000 nanodroplets via microfluidics that are then thermally-cycled (see Nathan *et al*.^37^ for details). Nanodroplets were then screened for fluorescence individually through the instrument for presence of target DNA fragments. Positive and negative droplets are counted to provide absolute quantification of target DNA.

### Estimating transport and retention metrics

In each of the 4 streams, we used the measured DNA concentrations to estimate transport lengths (*S*_*w*_, m)^39–41^, a quantitative metric representing the average distance eDNA travels in the water column before being physically or biologically retained. Transport length estimation is based on the assumption of a first order in space uptake process (i.e., *dN/dx*~−*N*), which results in an exponential decrease of concentration with downstream distance. We fit the eDNA concentration data from each experimental release to the following relationship: ln *N*_*x*_ = ln *N*_0_ − *ax*, where *N*_0_ and *N*_*x*_ are eDNA concentrations at the addition site (0 m) and *x* m downstream from the addition site; *a* is the per meter transport rate^41^ and the uptake length *S*_*w*_ is given by *a* ^−1^. As noted above, we also corrected measured eDNA concentrations for dilution using the conservative solute (NaCl), but these were minimal for these controlled short experimental stream reaches. Thus, we compared stream transport lengths across stream substrate types and used an ANCOVA to compare the slopes of the decline in eDNA along each stream reach^42^, using substrate treatment as the main effect and the stream sampling station characteristics (i.e., AFDM) as the covariate. While this test is typically performed to compare model intercepts and assumes equal slopes, we used the interpretation of an ANCOVA to determine any statistical difference between the slopes in each substrate treatment. This approach has been used previously to compare rates of both decomposition (*sensu* ^43^) as well as nutrient uptake (*sensu* ^44^) in differing streams.

The advantage of our approach lies in the ability to obtain both transport distances (expressed as an uptake length, S_w_) and depositional velocity (v_dep_) from our experimental data. Because mean transport distance is strongly influenced by discharge (*Q*, volume/time), and *Q* may vary among streams or within a stream over time, we calculated v_dep_, which removes the scaling effect of Q and allows direct comparison between streams with varying discharges. Thus, the transport distances measured through our experiments (S_w_) are not directly generalizable to other streams; however, when converted to a depositional velocity (v_dep_), they can be used to estimate transport distances of eDNA in systems of different size (as reflected in *Q*), and can thus be scaled to larger systems. When both parameters are considered together, a greater understanding of the factors controlling transport/retention in streams is possible, as these processes can be understood relative to stream size and *Q*. Therefore, from the estimates of *S*_*w*_, we also calculated retention as depositional velocity: *v*_dep_ (mm s^−1^) = (*Q*/*w)/S*_*w*_, where *Q* is stream discharge (in m^3^ s^−1^) and *w* is wetted channel width (in m)^45^, representing the apparent velocity at which a particle deposits from the water column to the benthic substrate. Calculation of *v*_dep_ allows for direct comparison of retention metrics across streams where discharge may vary by normalizing *S*_*w*_ for the effect of depth and velocity. We estimated discharge based on the mass balance of Cl^−^ in stream water, and widths and depths were averaged among 20 measured points along each stream reach.

### Estimating eDNA residence time

We plotted the breakthrough curve of ddPCR-quantified eDNA over time post-release (i.e., pump turned off) to estimate when the eDNA signal would disappear for each substrate treatment. We aimed to model the eDNA RTDs to estimate the hyporheic exchange rate (i.e., how quickly water is moving between the water column and the streambed) and truncation time (i.e., the time at which a positive eDNA detection is lost) to reflect resuspension that may have occurred over the stream reach^12^.

### Ethics

We declare no ethical considerations.

## Results

### Biofilm quantification

**Figure 3:**
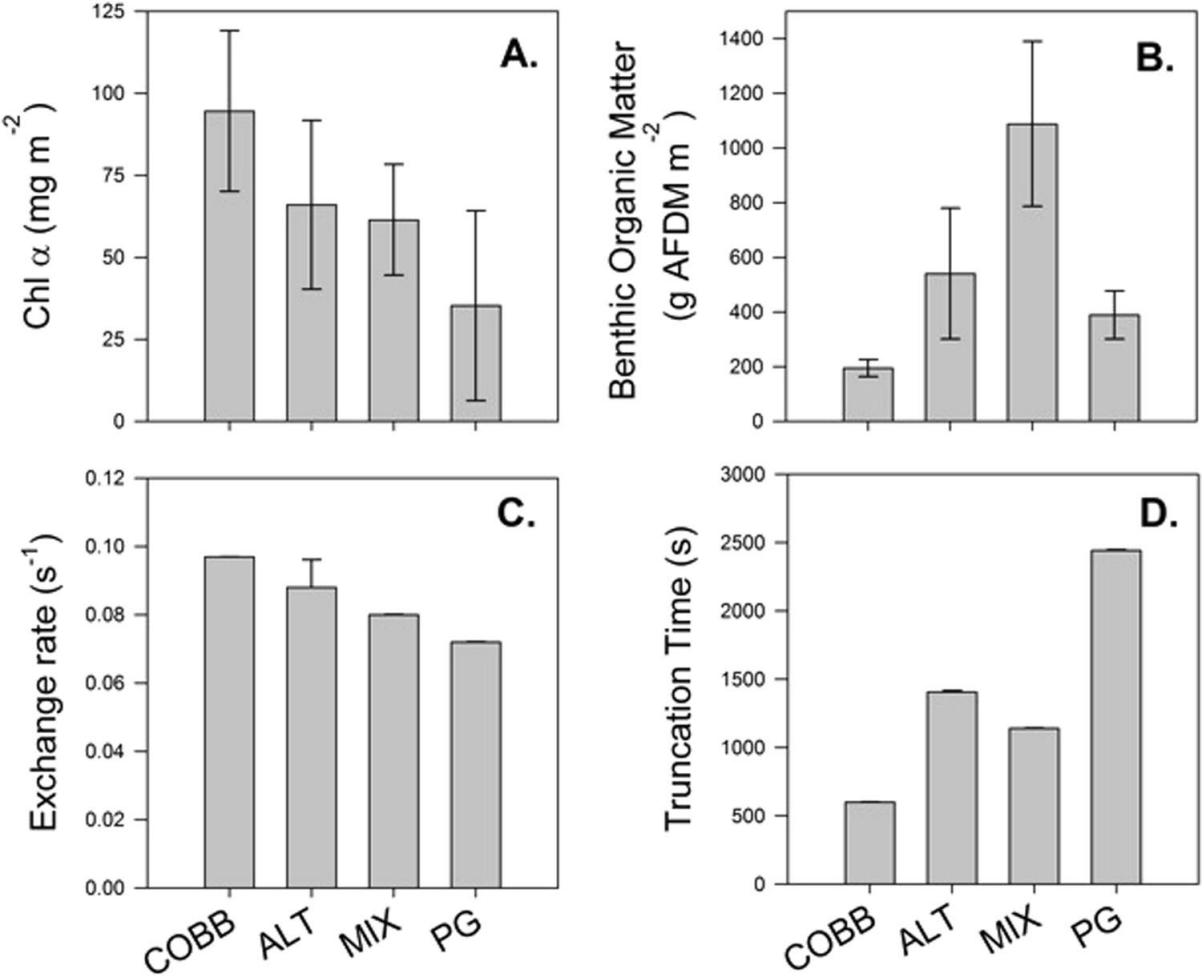
Quantification (±SE) of chlorophyll *a* (**A**), benthic organic matter (**B**), hyporheic exchange rate (**C**), and truncation time (**D**) for all LEEF streams. Data for panels C and D are from Aubeneau *et al*.^20^.

There was no statistical difference in chlorophyll *a* concentrations among streams (*ANOVA, F* = 1.125, *df* = 16, *p* = 0.34, Fig. 3A), and mean chlorophyll *a* was 61.4 mg/cm^2^. In contrast, we found that MIX had significantly higher AFDM (1.1 ± 0.7 g/cm^2^) (*ANOVA, F* = 3.76*, df* = 16*, p* = 0.032) than PG (0.4 ± 0.2 g/cm^2^), ALT (0.5 ± 0.5 g/cm^2^), and COBB (0.2 ± 0.1 g/cm^2^) (Fig. 3B). Given the differences in the organic matter content in each stream, we used AFDM as the covariate in the Analysis of Covariance (ANCOVA) described below. Hyporheic exchange rates (Fig. 3C) and truncation times (Fig. 3D) for conservative solutes are from Aubeneau *et al*.^20^ for the same streams.

### Setting the stage for the eDNA releases

**Figure 4:**
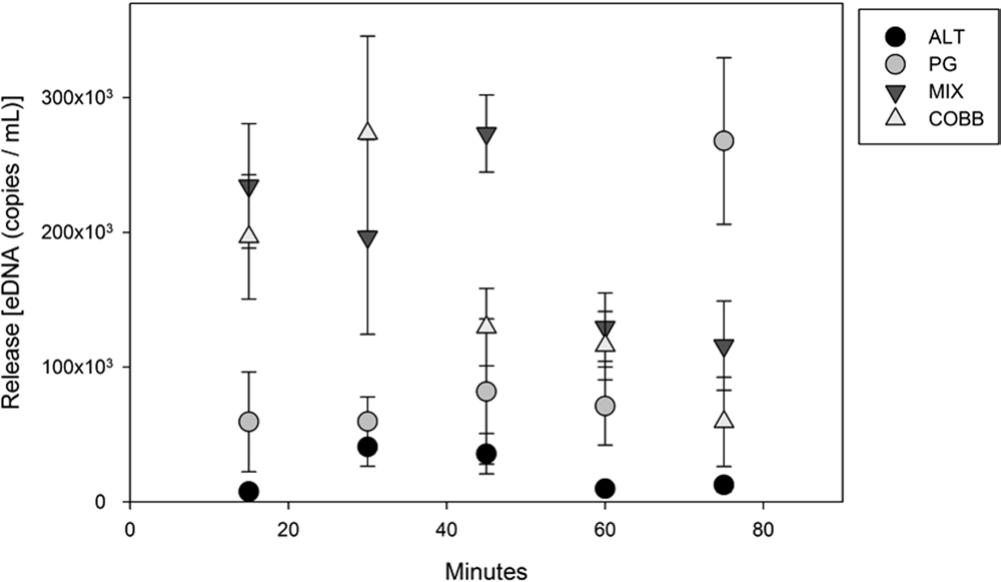
Concentrations of eDNA solution for over the course of the experimental release (n = 5 samples per stream; standard error bars represent 3 analytical replicates per sample). We found no statistical evidence of degradation in any stream (linear regression, p < 0.05).

We found a statistical difference in eDNA concentration between the release solution used in each of the four experimental eDNA releases (ANOVA, F^3, 16^, *p-value* = 0.009), with the lowest to highest mean concentrations going in order of: ALT, PG, COBB, and MIX (means found in Table 1). However, we conducted the experiments in ALT first, followed by MIX, PG, and COBB, which suggests that the variation in eDNA concentration in the release solution was not due to physical degradation. Additionally, there was also no statistical evidence of degradation in the carp eDNA release solution during each individual release (i.e., over ~4 hrs) throughout each experiment (linear regression, *p* > 0.05) (Fig. 4). Finally, we found no carp eDNA in any of the stream field controls (n = 20), cooler blanks (n = 4), or extraction blanks (n = 10).

**Table 1:** Parameter estimates for eDNA movement in LEEF streams using equations noted in methods.

Stream	Mean eDNA Release Solution ± SE (copies mL^−1^)	*Uptake Estimates*						
		Slope ± SE (k)	Transport Distance S_p_ ± SE (m)	Wetted Width (m)	Discharge Q (L sec^−1^)	Depositional Velocity v_dep_ ± SE (mm s^−1^)	*R* ^*2*^	*p*
COBB	21,274 ± 15673	—	—	0.56	1.87	—	—	—
ALT	107,919 ± 89863	−0.053 ± 0.01	18.9 15.9–23.3	0.62	2.1	0.146–0.214	0.64	<0.01
MIX	127,537 ± 87567	−0.106 ± 0.05	9.4 6.4–17.9	0.60	1.87	0.330 0.175–0.486	0.33	0.06
PG	189,966 ± 67271	−0.131 ± 0.02	7.7 6.7–9.1	0.59	2.1	0.464 0.392–0.535	0.76	<0.01

### Estimation of Transport (S_w_) and Retention (v_dep_)

**Figure 5:**
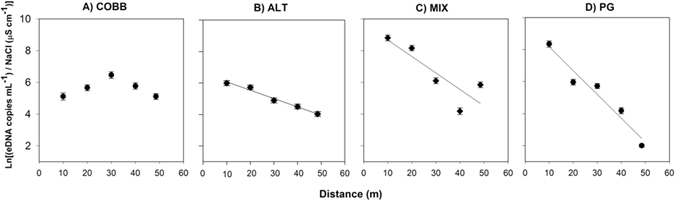
Regressions for COBB (**A**), ALT (**B**), MIX (**C**), and PG (**D**) natural-log transformed concentrations over distance used to estimate parameters reflected in Table 1. Each dot represents the mean of three replicate field samples ± SE bars.

Using the short-term eDNA addition approach, we quantified statistically-significant downstream declines in the mass of eDNA (as reflected in concentration) passing by each sampling station, allowing us to calculate the eDNA transport distance (S_w_) in 3 of the 4 streams (Fig. 5). We found that ALT had the longest measurable S_w_ of 21.7 m (*R* ^*2*^ = 0.74, *p* = 0.001, Fig. 4B), followed by MIX (MIX = 8.34 m, *R* ^*2*^ = 0.36, *p* = 0.06, Fig. 4C), and shortest S_p_ in PG at 6.7 m (*R* ^*2*^ = 0.76, *p* = 0.002, Fig. 4D). Finally, we saw no significant decline in eDNA along the experimental reach in COBB (Fig. 5A) and the observed concentration did not decrease monotonically with distance. Most importantly, we found a significant difference in S_w_ across the 3 streams with measurable eDNA retention, meaning that the slope of eDNA decline with distance was statistically different among streams with different substrate (ANCOVA, *p* < 0.05). We were also able to estimate depositional velocities, v_dep_, of eDNA in the 3 of 4 streams with significant retention. We found that ALT had the lowest v_dep_ of 0.154 mm/s, followed by MIX (v_dep_ = 0.407 mm/s) and the highest v_dep_ in PG at 0.462 mm/s. We were unable to estimate of v_dep_ in COBB because there was no detectable eDNA retention over the experimental 50 m reach.

### Resuspension pattern

**Figure 6:**
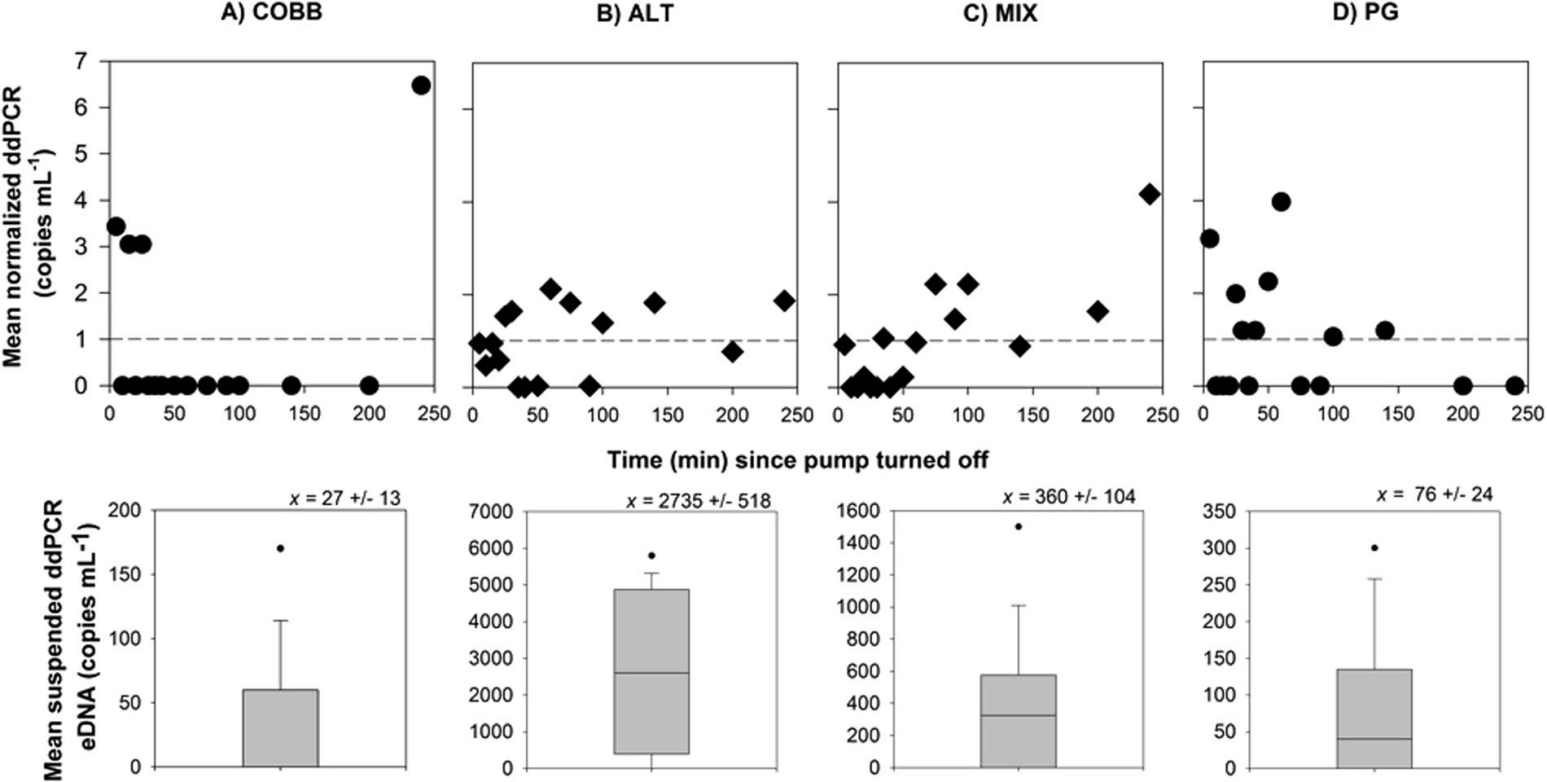
Normalized concentrations over time for COBB (**A**), ALT (**B**), MIX (**C**), and PG (**D**) once pump was turned off at *t* = *0* (i). All eDNA concentrations are normalized by the mean suspended concentrations in their respective stream to allow between stream comparisons (ii).

Result of the eDNA breakthrough curves using water samples analyzed using ddPCR showed stochastic, long retention patterns of low-concentration eDNA for 4 hrs after the end of each short-term eDNA addition experiments in all streams except COBB, and the pattern of eDNA resuspension varied with stream substrate type (Fig. 6). Given the stochastic nature of the eDNA release solution (Fig. 4), combined with the variable pattern in resuspension (Fig. 6), we were unable to estimate a mass balance of eDNA retained in each stream nor calculate eDNA RTDs. However, the low concentration in the “falling limb” samples relative to the influent release solution suggests that the majority of eDNA released into the stream was stored semi-permanently (i.e., beyond 4 hrs) in those streams where we were able to measure significant retention (i.e., PG, ALT, MIX). We did not run the experiment past 4 hours so that our transport, retention, and resuspension results would not be confounded by degradation, which can occur rapidly^46^.

## Discussion

### Estimating eDNA Retention

The use of eDNA for detection of vertebrates holds considerable promise for aquatic conservation biology^47–49^. Consideration of the mechanisms of eDNA transport and retention in flowing waters is critical for sampling design and spatial inference (i.e., distance upstream that a sample represents). We calculated depositional velocity (v_dep_), which removes the scaling effect of discharge and allows direct comparison between streams with varying flow. A key finding from this work is that eDNA is not transported uniformly in flowing water, but rather is differentially retained via interaction with benthic substrates and surface/subsurface interactions as water moves downstream (Fig. 1, Process 2). While conservative solute transport is dominated by advective and dispersive fluxes between the water column and the streambed (i.e., benthos), particles are also subject to the additional process of deposition and storage in the benthic region^50^. The interaction of suspended particles with the benthos leads to their retention in the streambed and decline in the overlying bulk water. In this study, we have demonstrated that benthic substrate plays an important role in eDNA transport, deposition, and subsequent retention, emphasizing the importance of hydrodynamic delivery of particles onto and into the streambed. Once deposited, or even during downstream travel, eDNA might also be subject to physical decay, sorption, consumption, or biological degradation, which represent additional removal processes that will occur over time.

Using the steady state release method to quantify eDNA movement, we were able to detect and quantify significant eDNA retention in streams with differing benthic substrate (Fig. 5). We were also able to quantify how the streambed influences downstream movement of eDNA, allowing us to fill a critical knowledge gap of how eDNA retention occurs along a stream reach. Importantly, this research also highlights the importance of considering the release concentration used in studies aiming at determining eDNA retention rates. In a similar study^12^, the mean release concentrations ranged from 345-1,040 copies mL^−1^, which did not reveal any differences between streams. However, using mean release concentrations ranging from 21,274 to 189,966 copies mL^−1^ in these experiments, we found that benthic composition had a significant impact on stream “retentiveness,” as eDNA retention rates were most related to general structural and hydrologic characteristics of the study reaches. Calculated retention rates were highly linked to instream RTD truncation time for conservative solutes, which expresses the length of time that a solute might remain in the bed and reflects the storage capacity or “retentiveness” of each stream, in addition to the exchange rates between the streambed and the bulk water column. As estimated by Aubeneau *et al*.^20^, in these streams finer homogenous substrate (PG) had very long instream residence and slower exchange rates, and coarser homogenous substrate (COBB) had the shortest instream residence and rapid exchange rates. The residence times of a conservative tracer in streams with complex (non-homogeneous) substrate structure (MIX, ALT) were intermediate between PG and COBB. Indeed, following these patterns, finer substrates (PG) tended to have faster eDNA removal, and thus shorter transport distances, than COBB, and no detectable removal was observed over the length of stream reach considered here for the coarser substrate. While we saw significant substrate-influenced retention, but the channels used at LEEF are concrete-lined and the bed exchange rates only represent the “micro” hyporheic zone caused by substrate porosity, and not true water loss into a lateral hyporheic zone. In real systems, retention may be further influenced by hyporheic exchange along a stream reach.

Our results provide support that the interaction between eDNA source and benthic composition can exert significant control over key eDNA retention processes in streams. In our experiments, stream flow and biology (i.e., biofilm) were similar among streams. Higher eDNA retention did not result solely from higher algal biomass or more organic matter, but rather, substrate-specific hydraulic differences accelerated particle deposition. For example, given that the ALT stream has 2 m sections of COBB and PG, one might expect that the results from ALT would fall between PG and COBB. For PG, our calculated regression slope of eDNA concentration over distance (*k*, 1 /m) was −0.131, but not measurable along the 50 m reach for COBB. We therefore estimated a COBB *k* = −0.0655, slightly higher than the observed value for ALT (*k* = −0.053). This discrepancy can be explained by the fact that each time the flow transitions between the COBB and PG sections, there is a “transition length” (essentially 10 times the average water depth, roughly 0.80 m in ALT) before the flow averages to behave as it would over the given substrate type. Therefore, if we assume that the system is only half as retentive over this length, an estimate closer to the observed value emerges. These findings are consistent with our intuitive predictions that finer substrate would retain particles of eDNA faster than coarser substrate (Fig. 1). However, the importance of physical habitat variability (i.e., the spatial variation of geomorphological properties within habitats) for particle retention and transport remains largely unexplored. Therefore, understanding the physical and biological variables that influence how eDNA particles are transported and retained in flowing waters remains essential for modeling efforts in complex systems.

### Estimating eDNA transport

In these experiments, we found the eDNA signal could become very low at relatively short distances downstream (e.g., S_w_ = 7.7 m, PG, Table 1) given our experimental conditions. Additionally, transport distances varied significantly based on structure and size of the benthic substrate (Fig. 1, Process 1). These results suggest that interactions with the streambed influence the mean distance that eDNA is transported, which could complicate interpretation of a positive result downstream. Calculated transport distance is not dependent on the initial concentration; however, we introduced a high concentration of eDNA into the system in order to accurately detect the decline of eDNA over distance in our experimental streams. This mean behavior would apply for single eDNA particles, irrespective of initial concentration, but the likelihood of getting a positive detection decreases as eDNA disperses spatially. Importantly, the transport distances (S_w_) calculated for the LEEF streams are inherently not generalizable to other streams, because the metric is dependent on stream size. However, when both parameters of v_dep_ and S_w_ are considered together, a greater understanding of the factors controlling transport and retention in streams is possible.

From the regression estimates for each substrate type, we can conservatively calculate the distance at which the qPCR-detectable eDNA signal would disappear given a steady source of eDNA particles and our qPCR detection limit of 3 copies mL^−1^. For ALT, the least retentive stream, the signal would decline after 87 m (95% CI from 73–107 m). For MIX, the signal would become undetectable at 72 m (95% CI from 50–136 m) and for PG, this would occur after 54 m (95% CI from 29–64 m). We were not able to measure significant transport distance in COBB, which implies that a longer experimental reach may be necessary to constrain the estimated transport distance of eDNA in system with larger substrate sizes. Given these estimates, a positive downstream eDNA signal suggests close proximity of a target organism, although the estimates of upstream distance were dependent on benthic substrate structure, along with characteristics of stream flow (i.e., width and depth, *sensu* Newbold *et al*.^45^ and eDNA sloughing rates^51^). The transport metric S_w_ is dependent on discharge (*Q*)^34, 45^, which was ~2 L/second in the experimental streams used in this study. In systems with higher *Q*, average eDNA transport distance would also increase. For example, mean stream discharge measured by Jane *et al*.^30^ was between 5–28 L/s; this study found positive eDNA signal over 200 m downstream. In even larger systems (>3000 L/s), eDNA was found over 12 km downstream^29^. Given the dependence of eDNA transport distance on *Q*, interpreting average transport lengths necessitates a clear understanding of stream or river size.

Our controlled, experimental streams allowed us to isolate the effect of substrate on eDNA transport and retention, but they are relatively uniform along each reach with median substrate diameter ranging from 0.5 cm (PG) to 5 cm (COBB). In contrast, in natural streams and rivers, reaches may be highly heterogeneous in substrate size and distribution, along with other physical characteristic such as flow variation in space and time. For example, the median substrate size (D_50_) of salmon spawning reaches in the Snake River, ID spatially varied from 1.3 cm to 6.9 cm^52^ with great variability around these values (ranging from very fine sediment <1 mm to boulders >25 cm). Given the increased complexity in natural systems, we suggest that average transport distances of eDNA are likely to change longitudinally along a reach, as habitat and substrate heterogeneity increase. Thus, in natural streams and rivers, detection is likely further complicated by continuous dilution of the eDNA signal with simultaneous displacement downstream^29^. Given that water column concentrations are typically low in natural streams and rivers, approaching eDNA detection limits especially when using conventional techniques (i.e., qPCR), estimating species distance upstream will become increasingly uncertain as species density decreases, sloughing rates decrease, or organism distance from sampling point increases. Additionally, eDNA is also subject to both biological or physical degradation while it is suspended during downstream transport or as it is removed from the water column^53^. Rates of eDNA degradation in flowing waters are not yet known, but are likely rapid given previous studies in standing waters.

While there is some evidence that eDNA can integrate biodiversity information in watersheds^14, 54^, the processes that drive single-species eDNA movement suggest that eDNA is not saturating downstream of its source. Rather, eDNA is being removed from the water column through processes such as settling^52^, destruction from physical forces and degradation^46, 55^, or, as shown in the present study, retained by the surrounding environment, including benthic substrate. These measured behaviors are further complicated as eDNA may be saltated along the streambed, in alternate deposition and resuspension events. Therefore, it will be challenging to simply back calculate species density or location from eDNA concentration data alone. While we do not have an estimation of the carp population in the pond water used in this experiment, we argue that in addition to calculating release rates from individual organisms^51^, accurate biomass prediction will require further information about environmental variables, including benthic structure and hydraulic behavior, due to the complexities of potential mechanisms driving eDNA transport and retention along a heterogeneous stream or river reach. By untangling the physical and ecological mechanisms governing eDNA transport, we can improve inferences about the presence of many imperiled and invasive species in lotic environments.

### eDNA Residence Patterns

We expected that eDNA from finer substrates would show more delayed resuspension, reflecting longer residence times in the streambed (i.e., heavier and more persistent tailing) after the eDNA source is removed. These expectations were developed following findings from a previous study^20^, where we measured the movement of rhodamine dye, a conservative tracer, in these same streams on a similar time scale (~3 hr). If eDNA behaved as we expected, the falling limb would begin to decay as a power law in time, reflecting retention and subsequent resuspension from the substrate, with concentration decaying consistently up to some cutoff time (i.e., truncation) after which all eDNA would be flushed from the system. The cutoff times estimated for detection of rhodamine in these streams were 600s (COBB), 1,141s (MIX), 1,406s (ALT) and 2,444s (PG), meaning that after this time no solute remains upstream. All of these times are far less than the time over which we measured the falling limbs for conservative tracers (4 hr = 14,400 seconds), so we believed that our 4 hr measurement time would be adequate in observing and estimating resuspension. However, we neither observe a monotonically decreasing concentration of eDNA over time nor a measurable cutoff point. Rather, the signal looks stochastic in nature (Fig. 6A–D), for all substrate types, with sudden increases or decreases in concentration and no immediately discernible relationship of concentration over time in these time series for the four experimental streams. This result is consistent with previous findings^12^. We do note, however, that COBB is an exception, where eDNA was rapidly flushed from the system.

In general, increased turbulence was associated with coarser substrate and faster exchange rates^20^ (Fig. 1, Process 3), which potentially influenced the rate of both deposition and subsequent resuspension. For example, higher turbulence in COBB likely resulted in little chance for deposition and more rapid flushing; in contrast, PG experiences less turbulence and likely higher deposition and retention. These results suggest that eDNA is not consistently being resuspended from the streambed at some deterministic rate as might be found with a conservative tracer or monodisperse particle, but rather at some stochastic time varying rate. In addition, this may be consistent with the suggestion of intermittency governing transport, where turbulent events of sufficient energy occur intermittently to re-suspend eDNA back into the water column for further transport downstream. Intermittency is ubiquitous in turbulent and porous media flows^56^ and is known to cause intermittency in particle transport in streams^57, 58^; we suggest here that this hypothesis is deserving of further study, given that resuspension of particles could influence later, downstream eDNA detection.

## Conclusions

Species monitoring has traditionally relied on physical identification and manual capture techniques, and aquatic species are notoriously difficult to detect with manual methods such as electroshocking and netting^59, 60^. Alternatively, traces of DNA (free DNA, cells, tissue, mucus, etc.) sloughed from an organism can remain in suspension and subsequently be collected in a water sample, revealing the presence of a target organism^3^. This is particularly important in highly susceptible ecosystems, such as networked rivers and streams, where early detection is essential for rapid management response^47, 61, 62^. While eDNA surveillance can be used with more accuracy than traditional methods for monitoring presence and absence of species^4^, the spatial and temporal distribution of eDNA in lotic systems has only recently been explored and demands further study^28^.

Our results show that eDNA movement is significantly more complicated than that of a conservative tracer or monodisperse solute of a particle, having both some level of apparently unpredictable behavior in the amount of eDNA recovered temporally downstream of the release and variability in retention across contrasting streambed substrates. In fact, eDNA displays multiple levels of complexity that standard modeling approaches for conservative tracers to not capture. While we were unable to constrain all aspects of eDNA transport behavior, the experiments described here offer significant progress towards incorporating realistic field conditions into a predictive framework for modeling eDNA transport and retention. These results alone offer a novel level of experimental control greater than most real-world field conditions and further highlights the potential difficulty in inferring species presence from positive and negative eDNA detection under lotic field conditions. Our results suggest that the use of eDNA sampling techniques in the field may necessitate more consideration in sampling effort and modeling to capturing the inherent variability and intermittent behavior of eDNA particles, as natural environment variability will likely be larger than in our any controlled experimental conditions.

## References

[1] Newbold JD, Thomas SA, Minshall GW, Cushing CE, Georgian T (2005). Deposition, benthic residence, and resuspension of fine organic particles in a mountain stream. Limnol. Oceanogr..

[2] Cole JJ (2007). Plumbing the global carbon cycle: Integrating inland waters into the terrestrial carbon budget. Ecosystems.

[3] Ficetola GF, Miaud C, Pompanon F, Taberlet P (2008). Species detection using environmental DNA from water samples. Biol. Lett.

[4] Jerde CL, Mahon AR, Chadderton WL, Lodge DM (2011). “Sight-unseen” detection of rare aquatic species using environmental DNA. Conserv. Lett..

[5] Dejean T (2011). Persistence of environmental DNA in freshwater ecosystems. PloS One.

[6] Rees HC, Maddison BC, Middleditch DJ, Patmore JR, Gough KC (2014). REVIEW: The detection of aquatic animal species using environmental DNA–a review of eDNA as a survey tool in ecology. J. Appl. Ecol..

[7] Goldberg CS, Pilliod DS, Arkle RS, Waits LP (2011). Molecular Detection of Vertebrates in Stream Water: A Demonstration Using Rocky Mountain Tailed Frogs and Idaho Giant Salamanders. PLoS One.

[8] Mächler E, Deiner K, Steinmann P, Altermatt F (2014). Utility of Environmental DNA for Monitoring Rare and Indicator Macroinvertebrate Species. Freshw. Sci.

[9] Simmons M, Tucker A, Chadderton WL, Jerde CL, Mahon AR (2016). Active and passive environmental DNA surveillance of aquatic invasive species. Can. J. Fish Aquat. Sci..

[10] Takahara T, Minamoto T, Yamanaka H, Doi H, Kawabata Z (2012). Estimation of fish biomass using environmental DNA. PLoS One.

[11] Takahara T, Minamoto T, Doi H (2013). Using Environmental DNA to Estimate the Distribution of an Invasive Fish Species in Ponds. PLoS One.

[12] Jerde CL (2016). The influence of stream bottom substrate on the retention and transport of vertebrate environmental DNA. Environ. Sci. Technol.

[13] Shogren AJ (2016). Modelling the transport of environmental DNA through a porous substrate using continuous flow-through column experiments. J. R. Soc. Interface.

[14] Deiner K (2016). Environmental DNA reveals that rivers are conveyer belts of biodiversity information. Nat. Commun..

[15] Leopold, L. B. Fluvial Processes in Geomorphology (1964).

[16] Banavar JR, Maritan A, Rinaldo A (1999). Nature.

[17] Rodríguez-Iturbe, I. & Rinaldo, A. *Fractal River Basins: chance and Self-Organization* Cambridge University Press (1997).

[18] Battin TJ, Kaplan LA, Newbold JD, Hansen CME (2003). Contributions of microbial biofilms to ecosystem processes in stream mesocosms. Nature.

[19] Arnon S, Marx LP, Searcy KE, Packman AI (2010). Effects of overlying velocity, particle size, and biofilm growth on stream subsurface exchange of particles. Hydrol. Process..

[20] Aubeneau AF, Hanrahan B, Bolster D, Tank JL, J. L (2014). Substrate size and heterogeneity control anomalous transport in small streams. Geophys. Res. Lett..

[21] Cushing CE, Minshall GW, Newbold JD (1993). Fine particulate organic matter transport dynamics in two Idaho streams. Limnol. Oceanog..

[22] Mullholland PJ (2008). Stream denitrification across biomes and its response to anthropogenic nitrate loading. Nature.

[23] Drummond JD, Aubeneau AF, Packman AI (2014). Stochastic modeling of fine particulate organic carbon dynamics in rivers. Wat. Resour. Res..

[24] Aubeneau AF, Hanrahan B, Bolster D, Tank JL (2016). Biofilm growth in gravel bed streams controls solute residence time distributions. Geophys. Res. Lett..

[25] Jerde CL, Mahon AR (2015). Improving confidence in environmental DNA species detection. Mol. Ecol. Resour..

[26] Barnes MA, Turner CR (2016). The ecology of environmental DNA and implications for conservation genetics. Conserv. Genet..

[27] Turner CR (2014). Particle size distribution and optimal capture of aqueous macrobial eDNA. Methods Ecol. Evol.

[28] Wilcox TM, McKelvey KS, Young MK, Lowe WH, Schwartz MK (2015). Environmental DNA particle size distribution from Brook Trout (Salvelinus fontinalis). Conservation Gen. Resour..

[29] Deiner K, Altermatt F (2014). Transport distance of invertebrate environmental DNA in a natural river. PLoS ONE.

[30] Jane SF (2015). Distance, flow and PCR inhibition: eDNA dynamics in two headwater streams. Mol. Ecol. Resour..

[31] Laramie MB, Pilliod DS, Goldberg CS (2015). Characterizing the distribution of an endangered salmonid using environmental DNA analysis. Biol. Conserv..

[32] Spear SF, Groves JD, Williams LA, Waits LP (2015). Using environmental DNA methods to improve detectability in a hellbender (Cryptobranchus alleganiensis) monitoring program. Biol. Conserv..

[33] APHA. Standard Methods for the Examination of Water and Wastewater, 20th ed., American Public Health Association (1998).

[34] Stream Solute Workshop (1990). Concepts and methods for assessing solute dynamics in stream ecosystems. J. N. Am. Benth. Soc.

[35] Hall RO, Tank JL (2003). Ecosystem metabolism controls nitrogen uptake in streams in Grand Teton National Park, Wyoming. Limno. Ocean..

[36] Renshaw MA, Olds BP, Jerde CL, McVeigh MM, Lodge DM (2015). The room temperature preservation of filtered environmental DNA samples and assimilation into a Phenol‐Chloroform‐Isoamyl alcohol DNA extraction. Mol. Ecol. Res..

[37] Nathan LM, Simmons M, Wegleitner BJ, Jerde CL, Mahon AR (2014). Quantifying environmental DNA signals for aquatic invasive species across multiple detection platforms. Environ. Sci. Technol..

[38] Doi H (2015). Droplet digital polymerase chain reaction (PCR) outperforms real-time PCR in the detection of environmental DNA from an invasive fish species. Environ. Sci. Technol..

[39] Miller J, Georgian T (1992). Estimation of fine particle transport in streams using pollen as a seston analog. J. North Am. Benthol. Soc..

[40] Thomas SA (2001). The influence of particle size on seston deposition in streams. Limnol. Oceanogr..

[41] Paul MJ, Hall RO (2002). Particle transport and transient storage along a stream-size gradient in the Hubbard Brook Experimental Forest. J. North Am. Benthol. Soc..

[42] Kleinbaum, D.G., Kupper, L.L., Muller, K.E. *Applied Regression Analysis and Other Multivariate Methods* Kent Publishing Co., Boston MA (1988).

[43] Hagen EM, Webster JR, Benfield EF (2006). Are leaf breakdown rates a useful measure of stream integrity along an agricultural landuse gradient?. J. N. Am. Benthol. Soc..

[44] Tank JL, Rosi-Marshall EJ, Baker MA, Hall RO (2008). Are rivers just big streams? A pulse method to quantify nitrogen demand in a large river. Ecology.

[45] Newbold JD, Elwood JW, O’Neill RV, Van Winkle W (1981). Measuring nutrient spiraling in streams. Can. J. Fish Aquat. Sci..

[46] Barnes MA (2014). Environmental conditions influence eDNA persistence in aquatic systems. Environ. Sci. Technol..

[47] Lodge DM (2012). Conservation in a cup of water: estimating biodiversity and population abundance from environmental DNA. Mol. Ecol..

[48] Bohmann K (2014). Environmental DNA for wildlife biology and biodiversity monitoring. Trends Ecol. Evol..

[49] Lawson Handley L (2015). How will the ‘molecular revolution’ contribute to biological recording?. Biol. J. Linn. Soc..

[50] Ren J, Packman AI (2002). Effect of Background Water Composition on Stream-Subsurface Exchange of Submicron Colloids. J. Environ. Eng.-ASCE.

[51] Klymus KE, Richter CA, Chapman DC, Paukert C (2015). Quantification of eDNA shedding rates from invasive bighead carp *Hypophthalmichthys nobilis* and silver carp *Hypophthalmichthys molitrix*. Biol. Conserv..

[52] Platts, W. S., Shirazi, M. A. & Lewis, D. H. Sediment particle sizes used by salmon for spawning, and methods for evaluation. *Rep. EPA-600/3-79-043*, 32 (1979).

[53] Eichmiller JJ, Miller LM, Sorensen PW (2016). Optimizing techniques to capture and extract environmental DNA for detection and quantification of fish. Mol. Ecol. Resour..

[54] Olds BP (2016). Estimating species richness using environmental DNA. Ecol. Evol..

[55] Turner CR, Uy KL, Everhart RC (2015). Fish environmental DNA is more concentrated in aquatic sediments than surface water. Biol. Conserv..

[56] De Anna P (2013). Flow intermittency, dispersion, and correlated continuous time random walks in porous media. Phys. Rev. Lett..

[57] Singh A, Fienberg K, Jerolmack DJ, Marr J, Foufoula-Georgiou E (2009). Experimental evidence for statistical scaling and intermittency in sediment transport rates. J. Geophys. Res..

[58] Escauriaza E, Sotiropoulos F (2011). Reynolds number effects on the coherent dynamics of the turbulent horseshoe vortex system. Flow Turbul. Combust..

[59] Murphy, B. R., & Willis, D. W. *Fisheries techniques* (*2nd ed*.). American Fisheries Society (1996).

[60] Hauer, F. R., & Lamberti, G. A. (Eds.). *Methods in stream ecology*. Academic Press (2011).

[61] Nathan LR, Jerde CL, McVeigh M, Mahon AR (2014). An assessment of angler education and bait trade in the Laurentian Great Lakes. Manag. Biol. Invasion.

[62] Simmons M, Tucker A, Chadderton WL, Jerde CL, Mahon AR (2015). Active and passive environmental DNA surveillance of aquatic invasive species. Can. J. Fish Aquat. Sci..

